# Thyroid Storm-induced Takotsubo Cardiomyopathy Presenting as Acute Chest Pain: A Case Report

**DOI:** 10.5811/cpcem.2021.4.52005

**Published:** 2021-10-05

**Authors:** Brayden Ashdown, Emilie Calvello Hynes

**Affiliations:** * Denver Health, Department of Emergency Medicine, Denver, Colorado; † University of Colorado School of Medicine, UC Health, Department of Emergency Medicine, Aurora, Colorado

**Keywords:** Thyroid storm, Takotsubo, cardiomyopathy, chest pain, case report

## Abstract

**Introduction:**

Stress-induced cardiomyopathy is a rare but serious cause of chest pain, which in recent studies has been shown to carry a similar in-hospital mortality to acute ST-elevation myocardial infarction. The pathophysiology of the disease is thought to be secondary to dysregulated catecholamine effects on myocardium.

**Case Report:**

We present a case of a previously healthy female without known thyroid disease who presented to the emergency department for acute chest pain and was found to have thyroid storm-induced cardiomyopathy in a typical stress-induced cardiomyopathy pattern without evidence of coronary disease on catheterization.

**Conclusion:**

Thyrotoxicosis can cause dysregulation of catecholamines and is a rare cause of stress-induced cardiomyopathy. It requires distinct therapies and should be considered by emergency physicians in the workup of acute chest pain with concern for stress-induced cardiomyopathy.

## INTRODUCTION

Stress-induced cardiomyopathy (also known as Takotsubo cardiomyopathy) is an acute onset cardiomyopathy of unclear etiology.^1^–^4^ There are many proposed mechanisms; however, despite significant research the underlying pathophysiology has not yet been clearly established. The diagnosis is made largely based on clinical history and imaging studies.^2^,^5^ This disease is most commonly seen in post-menopausal women after an acute psychosocial stressor, but case reports exist for a variety of preceding medical conditions, including anaphylaxis, pancreatitis, pheochromocytoma, tricyclic overdose, and even lightning strike.^4^ Although the condition is rarely seen, the pathophysiologic basis for hyperthyroid-induced stress cardiomyopathy aligns with current understanding of the disease process.^1^,^2^

The etiology of heart failure in this case is fundamentally different from high output failure more commonly seen in thyrotoxic states, which is driven primarily by decreased systemic vascular resistance.^4^ Thyrotoxicosis as an etiology of stress-induced cardiomyopathy is important to recognize because it is easily treated; however, the therapies are distinct from what are typically used in stress-induced cardiomyopathy. Lack of prompt treatment may portend a worse prognosis.^6^

## CASE REPORT

**Image 1:**
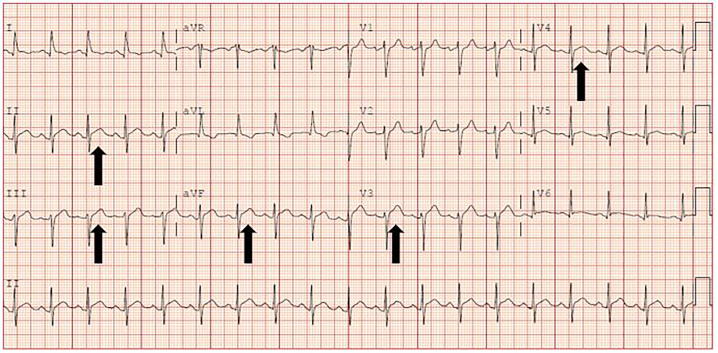
Presenting electrocardiogram, read by physician as 1 millimeter of ST elevation, most prominent in inferior and anterior leads (arrows).

A 61-year-old female with history of pulmonary embolism (PE), not on anti-coagulation, presented to the emergency department (ED) with pressure-like chest pain and associated dyspnea starting approximately eight hours prior to presentation. Her electrocardiogram on arrival had one-millimeter ST elevations in anterior and inferior leads, and the triage physician activated the cardiac alert system (Image 1).

On arrival to the resuscitation room in the ED, she was afebrile, had a normal oxygen saturation on room air, a blood pressure of 151/85 millimeters of mercury and a heart rate of 142 beats per minute. The patient reported ongoing chest pain. She was given 325 milligrams of aspirin, and the interventional cardiologist was consulted. The patient was awake and alert and was answering questions appropriately. She reported that her prior PE had been provoked in the setting of childbirth over 20 years earlier but remained anticoagulated until four years prior to her current presentation when she discontinued, after a discussion of risks and benefits with her primary care physician. She had no other significant medical history and was not taking any medications regularly. Review of systems was notable only for a recent diagnosis of shingles, currently on antiviral medications.

**Image 2:**
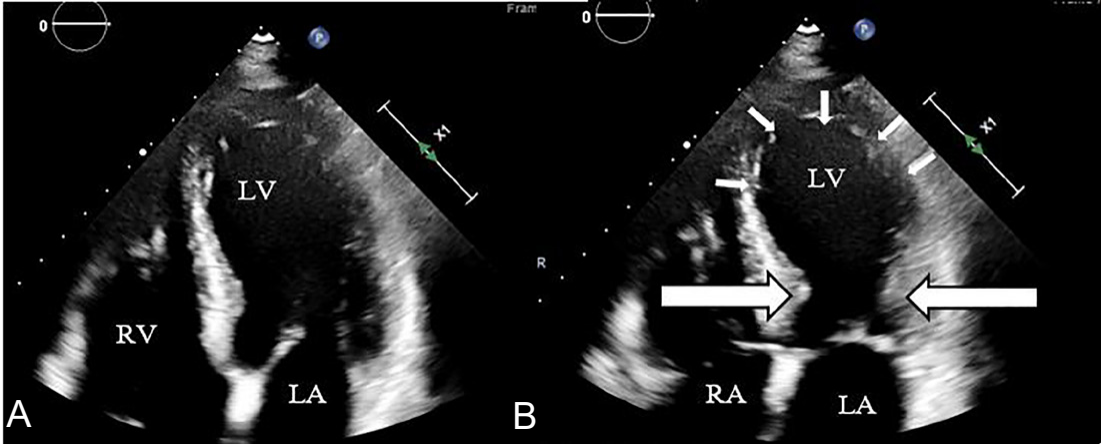
Point-of-care ultrasound images in the apical four-chamber view: A) diastole and B) systole demonstrating normal to hypercontraction of the base of the left ventricle (large arrows) and a hypodynamic apex (small arrows). *LV,* left ventricle; *RV,* right ventricle; *LA,* left atrium; *RA,* right atrium.

Point-of-care ultrasound was performed, which demonstrated markedly reduced ejection fraction with apical hypokinesis, no pericardial effusion, no evidence of right ventricular strain, no aortic flap or dilation, and no evidence of deep vein thrombosis in bilateral lower extremities (Video). Due to the apical predominant dysfunction, the patient was asked about recent psychosocial life-stressors, which she denied (Image 2). She had a single view chest radiograph which showed no mediastinal widening, evidence of pulmonary edema, or pneumonia. Initial laboratory data were unremarkable with the exception of a potassium of 3.4 millimoles (mmol) per liter (L) (reference range: 3.5–5.1 mmol/L), a brain natriuretic peptide level of 227 picograms per milliliter (pg/mL) (0–100 pg/mL), and a troponin of 6.0 nanograms (ng)/mL (<=0.04 ng/mL). Given ongoing chest pain and electrocardiographic findings, the decision was then made in conjunction with the interventional cardiology team to take the patient to the cardiac catheterization lab to rule out an ischemic etiology, although stress-induced cardiomyopathy was a leading diagnosis on the differential.

**CPC-EM Capsule**

What do we already know about this clinical entity?

*Stress-induced cardiomyopathy is an uncommon cause of chest pain that can present with ST-segment elevation mimicking myocardial infarction.*

What makes this presentation of disease reportable?

*This patient had thyroid storm, a rare cause of stress-induced cardiomyopathy, requiring distinct therapies for management of hyperthyroidism and heart failure.*

What is the major learning point?

*Severe hyperthyroidism is a rare cause of stress-induced cardiomyopathy.*

How might this improve emergency medicine practice?

*In evaluation of stress-induced cardiomyopathy, consider hyperthyroidism as a possible etiology. Ultrasound is a rapid means to narrow the differential in chest pain.*

Coronary angiography showed no evidence of coronary artery disease. The patient was subsequently transferred to the cardiac intensive care unit (CICU) for ongoing management of acute cardiomyopathy. At time of admission to the CICU, the patient’s dyspnea had worsened, and she had a three to four liter oxygen requirement. While in the catheterization lab, thyroid studies resulted with an undetectable thyroid stimulating hormone and a free thyroxine (T4) level of 5.75 ng/dL (0.89 – 1.76 ng/dL). Endocrinology was consulted to assist in management of a presumed thyroid storm. Further history revealed no prior history of known thyroid disease, but mild symptoms of thyrotoxicosis including weight loss (22 pounds in the prior month) and new intermittent tremor over the preceding several months.

Due to concerns that thyroid storm was contributing to heart failure symptoms, the inpatient team started propylthiouracil at 400 milligrams (mg) every six hours, potassium iodide oral solution (SSKI) five drops every six hours, hydrocortisone 100 mg intravenously every eight hours, and an esmolol infusion (to allow careful titration given reduced ejection fraction). Heart rate, palpitations, and dyspnea improved with treatment of thyroid storm. During hospitalization, thyroid stimulating hormone receptor antibody, thyroglobulin antibody, and thyroid peroxidase antibody levels were found to be elevated, consistent with Graves’ disease.^6^

## DISCUSSION

Stress-induced cardiomyopathy was traditionally thought to be a relatively benign condition in which appropriately treated patients rarely die and typically completely recover cardiac function in 6–8 weeks. Unfortunately, more recent studies have demonstrated an early in-hospital mortality of around 4–5%, a figure “comparable to that of ST-segment elevation myocardial infarction [STEMI] in the era of primary percutaneous coronary interventions.”^2^ These findings highlight the importance of early recognition and treatment, as well as a more complete understanding of the underlying pathophysiology. Although many mechanisms have been proposed, the leading theories surround the dysfunctional effects of catecholamines on cardiac myocytes.^1^–^2^,^5^ There is evidence of not only pathologic upregulation of catecholamine and neuropeptide levels in patients with stress-induced cardiomyopathy when compared to patients with STEMI but also abnormal, apex-predominate, sympathetic innervation. The proposed mechanisms include direct toxic effects on cardiac myocytes, cyclic adenosine monophosphate mediated calcium toxicity, and cytokine mediated inflammation.^1^,^2^ These mechanisms underpin the rationale for beta-blocker therapy, which has been shown to be very effective in recovery of function in stress-induced cardiomyopathy.

By similar mechanistic pathways, hyperthyroidism can affect a similar physiologic state to catecholamine excess and, therefore, lead to stress-induced cardiomyopathy. Thyroid hormone can both directly upregulate beta-adrenergic receptors on myocardial tissue and sensitize those receptors to an exaggerated response to endogenous catecholamines.^1^,^7^ Thyroid hormones likely also have a direct effect on myocytes based on animal data showing cardiovascular response to hyperthyroid states in knockout mice lacking all beta receptors.^1^, ^8^ Although the condition is rare, a review in *Thyroid* found 14 case reports of hyperthyroid-induced stress cardiomyopathy. Prompt treatment of hyperthyroid states led to complete recovery in the cases that those authors were able to follow.^1^

A second, more generally applicable learning point from this case is the utility of point-of-care echocardiography in undifferentiated patients with chest pain. Chest pain is the second most common chief complaint among patients presenting to EDs in the United States.^5^ While most patients who present with chest pain are ultimately diagnosed with benign etiologies of their pain,^4^,^5^ there are several life-threatening causes, which require time-consuming, expensive, and potentially harmful diagnostic tests. Narrowing the differential diagnosis, which includes aortic dissection, acute coronary syndrome, pericardial effusion and tamponade, massive PE and acute decompensated heart failure, can pose a diagnostic challenge where time to therapy can make a difference in morbidity and mortality. This case demonstrates the utility of point-of-care ultrasound in quickly and efficiently narrowing that differential.

In this case, PE was a significant concern given the patient’s tachycardia, dyspnea and history of prior PE, not currently on anticoagulation. Point-of-care echocardiogram and deep vein thrombosis ultrasound not only indicated PE was less likely but also showed reduced likelihood of dissection (normal appearing aortic root), pericardial effusion/tamponade (no effusion), and acute decompensated heart failure (no B-lines).^9^ Although cardiac catheterization is still standard of care for patients presenting with more clear cases of stress-induced cardiomyopathy to rule out ischemic disease, characteristic point-of-care echocardiography can also impact the timing and subsequent resource utilization as well as urgency of transfer to a cardiac catheterization center. In this case the interventional team was already on site; so the patient was taken directly to the catheterization suite for what was already determined to be a non-emergent evaluation of her coronary arteries.

## CONCLUSION

Stress-induced cardiomyopathy is an uncommon cause of acute chest pain presenting to the ED, but a cause that carries an in-hospital mortality similar to acute ST-elevation myocardial infarction. The dysfunctional catecholamine regulation seen in thyroid storm can lead to a similar physiologic state as acute stress from medical illness or psychosocial stressors and lead to stress-induced cardiomyopathy. The prompt identification and treatment of this unique cause of chest pain allows for interventions that may prevent ongoing damage to the myocardium and provide the best chance at recovery for patients. Point-of-care ultrasound can assist in early identification due to the characteristic findings seen in stress-induced cardiomyopathy and the absence of findings consistent with alternative etiologies of chest pain.

## Supplementary Information

Video. Point-of-care ultrasound video throughout the cardiac cycle in the apical four chamber view, demonstrating normal to hypercontraction of the base of the left ventricle and a hypo dynamic apex.
